# Non‐discontinuation of antiseizure medication in seizure‐free epilepsy patients

**DOI:** 10.1111/ene.16160

**Published:** 2023-11-28

**Authors:** Jakob I. Doerrfuss, Thea Hüsing, Luise Graf, Maria Ilyas‐Feldmann, Martin Holtkamp

**Affiliations:** ^1^ Charité—Universitätsmedizin Berlin, Corporate Member of Freie Universität Berlin and Humboldt‐Universität zu Berlin, Department of Neurology with Experimental Neurology Berlin Germany; ^2^ Center for Stroke Research Berlin Berlin Germany; ^3^ Epilepsy‐Center Berlin‐Brandenburg Institute for Diagnostics of Epilepsy Berlin Germany

**Keywords:** drugs, epilepsy, pharmacotherapy, prognosis, seizure

## Abstract

**Background and purpose:**

In patients with epilepsy and sustained seizure freedom, guidelines recommend considering discontinuation of antiseizure medication (ASM) based on shared decision‐making. This study aims to identify factors associated with non‐discontinuation of ASM in seizure‐free patients.

**Methods:**

Retrospective data from three sites of an academic outpatient clinic were analyzed. Adult patients with epilepsy who have been seizure‐free for ≥24 months on ASM monotherapy were included. The primary end‐point was non‐discontinuation of ASM, defined as no discontinuation or no dose reduction of ≥25% at the last outpatient clinic visit in the ultimate seizure‐free interval. Secondary end‐points included frequency of discussion on discontinuation attempts between patients and physicians, adherence to ASM discontinuation decisions, and post‐discontinuation seizure outcomes.

**Results:**

Out of 338 included patients, 81.7% did not discontinue ASM and did not reduce its dose, 11.5% discontinued ASM and 6.8% had a significant dose reduction. Factors independently associated with non‐discontinuation of ASM were history of focal to bilateral or generalized tonic–clonic seizures (odds ratio [OR] 2.33, 95% confidence interval [CI] 1.08–5.06), history of breakthrough seizures (OR 3.32, 95% CI 1.10–10.04), history of failed attempts to discontinue or reduce the ASM dose (OR 4.67, 95% CI 1.03–21.11) and higher ASM load at the index visit (OR 6.10, 95% CI 2.09–17.78). Discontinuation attempts were made during the entire period of seizure freedom and were most commonly undertaken ≥10 years after the last seizure.

**Conclusions:**

This study provides insights into factors associated with the shared decision‐making process regarding ASM discontinuation in seizure‐free patients and highlights the importance of considering individual patient characteristics and seizure history.

## INTRODUCTION

In people with epilepsy, antiseizure medication (ASM) is the main pillar of treatment. Approximately 60%–85% of patients achieve sustained seizure freedom [1, 2]. When patients have become seizure‐free for some time, the question arises whether ASM may be discontinued. Typically, physicians recommend discontinuing ASM or reducing its dose significantly only when they estimate the risk for seizure recurrence to be low [3, 4]. Several factors have been described to be associated with seizure remission after ASM discontinuation [5, 6, 7].

As duration since last seizure is one of the strongest predictors for remaining seizure‐free, ASM discontinuation is usually recommended if the patient has been without seizures for a minimum of 2 years [3, 4]. However, a significant proportion of patients remain on ASM long beyond that time [8]. As long‐term ASM therapy may be associated with significant adverse effects [9, 10], which reduce quality of life [11, 12] and impose financial burdens on the healthcare system [13], it is important to identify factors associated with lack of discontinuation or significant dose reduction of ASM.

Previous studies on seizure‐free patients have shown that ASM discontinuation is discussed in only 30%–50% of outpatient visits [8, 14]. In a recent study on a heterogeneous patient population with focus on the first decade of seizure freedom including children, adolescents and adults treated in monotherapy and polytherapy, the decision to attempt ASM discontinuation was independently associated with four variables: longer duration of seizure freedom, treatment with an older generation ASM, self‐limiting epilepsy syndrome and lower estimated risk of seizure recurrence as calculated with the antiepileptic drug withdrawal risk retrieval tool [6, 8].

Nevertheless, there are gaps in the evidence regarding ASM discontinuation practices in outpatient epilepsy clinics. It remains unstudied how previous unsuccessful discontinuation attempts and breakthrough seizures influence ASM discontinuation decisions. Furthermore, open questions persist regarding timing of ASM discontinuation, particularly concerning discontinuation after more than 10 years of seizure freedom. It is possible that the probability of discontinuing ASM or reducing the dose is lower after a long time of seizure freedom as either the patients have made a final decision regarding continuation of ASM therapy or the physicians have given up on suggesting ASM discontinuation.

In the light of these considerations, this study aims to examine factors associated with non‐discontinuation (i.e., lack of either discontinuation or dose reduction of at least 25%) of ASM monotherapy in seizure‐free patients.

## METHODS

### Data source and participants

Data from the three sites of the epilepsy outpatient clinic of the Department of Neurology at Charité—Universitätsmedizin Berlin in Germany were retrospectively analyzed. Approval for the study was granted by the local ethics committee (EA2/181/20). The study conforms with the World Medical Association Declaration of Helsinki. Since this study was retrospective, the requirement for individual patient informed consent was waived.

All adult patients with a diagnosis of epilepsy according to the International League Against Epilepsy [15], who had been seizure‐free for at least 24 months on ASM monotherapy, were included. Patients who were pregnant in the respective seizure‐free interval, patients in whom resective epilepsy surgery had been performed, patients with ASM polytherapy, patients in whom the seizure‐free interval had occurred before treatment in our outpatient clinics as well as patients with insufficient documentation were excluded.

All visits to the outpatient clinics from January 2010 until 30 September 2022 were potentially eligible for inclusion.

**FIGURE 1:**
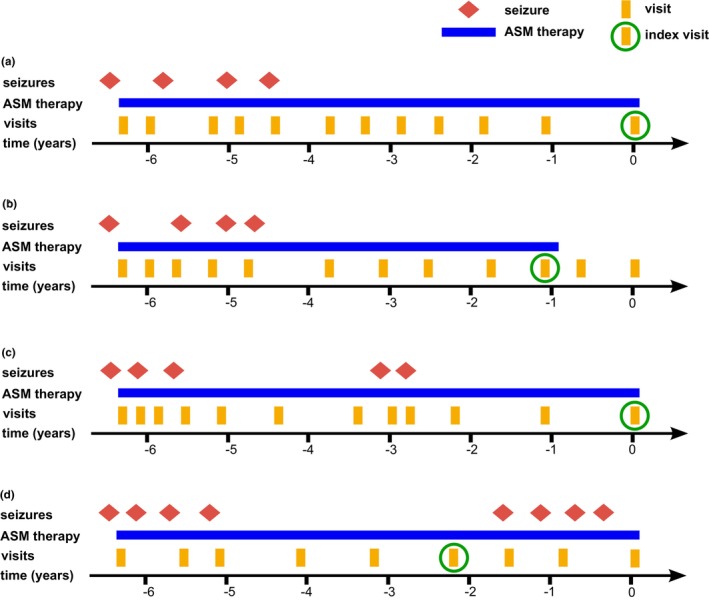
Definition of index visits. ASM, antiseizure medication. This figure shows examples of index visits in different exemplary patients. (a) This patient has been seizure‐free for approximately 4 years and has not yet discontinued ASM therapy. The index visit is the last visit in the seizure‐free interval. (b) This patient has discontinued ASM therapy after a seizure‐free interval of approximately 4 years. The index visit is the last visit in the seizure‐free interval where the patient still received ASM. (c) This patient has two seizure‐free intervals >24 months. Only the last visit in the last seizure‐free interval is considered the index visit. (d) This patient had a seizure‐free interval of approximately 3.5 years but now again has seizures. The last visit in the seizure‐free interval is considered the index visit.

For each patient fulfilling the inclusion criteria, retrieval and analysis of data refer to the index visit. The index visit was defined as the last visit to the epilepsy outpatient clinic in the ultimate seizure‐free interval of >24 months in which the patient was still taking one ASM. If a patient had more than one seizure‐free interval, the ultimate interval was analyzed. This was done to prevent any potential distortion of our findings pertaining to demographic factors. Figure 1 shows examples of the application of our definition of index visits in different illustrative patients.

### Primary and secondary end‐points

The primary end‐point of our study was non‐discontinuation of ASM (i.e., not discontinuing ASM or not reducing the dose by at least 25%) at the index visit. Our study goal was to identify factors that are associated with the primary end‐point and to analyze rate of non‐discontinuation of ASM according to duration of seizure freedom.

Secondary end‐points were (i) whether the topic of reducing or discontinuing ASM was actually addressed by the treating physician at the index visit (irrespective of the final decision), (ii) whether a shared decision by physician and patient to reduce or discontinue ASM was followed by the patient and (iii) to what extent patients remained seizure‐free after discontinuation of ASM.

### Data extraction

The data were retrieved from the patients' electronic medical records examining the following demographic and clinical parameters: sex, duration of epilepsy before remission, duration of seizure freedom, epilepsy type, number of all ASMs taken so far (including current ASM), number of previous seizures (1–9 seizures vs. ≥10 seizures), family history of epilepsy, history of febrile seizures, history of status epilepticus, history of breakthrough seizures (defined as seizure recurrence after 12 months of seizure freedom without reduction of ASM dose in the prior 3 months) and history of failed attempts to reduce or discontinue ASM.

In addition, the following data specific to the index visit were retrieved: age at index visit, duration of epilepsy and duration of seizure freedom at the time of the index visit, Liverpool Adverse Effects Profile score [16], decision of physicians regarding discontinuation or dose reduction of ASM as well as specific ASM drug and dose. Individual ASM drug loads (i.e., a normalized ASM dose for each patient) were calculated according to the 2020 World Health Organization Center for Drug Statistics Methodology ATC/DDD Index with the formula “individual daily ASM dosage divided by defined daily dose (DDD)”. ASMs were also categorized based on their approval dates. Carbamazepine, ethosuximide, phenobarbital, phenytoin, primidone and valproic acid were defined as first‐generation ASMs.

In patients in whom the index visit was not the same as the last visit to the outpatient clinic (i.e., cases where ASM had been discontinued, and patients in whom seizures had re‐occurred after the index interval; for case examples, see Figure 1b,d), data on seizure outcome since the index visit and adherence to the decision to continue or discontinue ASM therapy were also retrieved.

Taking together all available data, 2‐ and 5‐year seizure recurrence risk after possible ASM discontinuation was calculated according to the antiepileptic drug withdrawal risk retrieval tool (http://epilepsypredictiontools.info/aedwithdrawal) [6]. The recurrence risks were calculated using a Microsoft Excel script provided by its authors.

### Statistical analysis

Categorical variables were expressed as frequencies and were analyzed with Pearson's chi‐squared tests. Due to a non‐normal distribution as checked in the Kolmogorov–Smirnov test, continuous variables were expressed as median and interquartile range (IQR) and were analyzed using Mann–Whitney *U* tests. Confidence intervals (CIs) of frequencies were calculated using the exact Clopper‐Pearson method. A significance level of *p* < 0.05 was considered statistically significant.

Adjusted multiple logistic regression analysis (inclusion method: enter) was conducted to calculate the odds ratio (OR) with 95% CI in order to identify variables independently associated with consensus to not discontinue or reduce ASM. Age, sex and variables with *p* values <0.25 in univariate analysis were included in our logistic regression analysis. The calculated 2‐year and 5‐year recurrence risks were not included in our model to avoid multicollinearity as the calculation of these risks encompassed several parameters already included in the logistic regression analysis. All statistical analyses were performed using SPSS Statistics, version 28.

## RESULTS

### Study population

**FIGURE 2:**
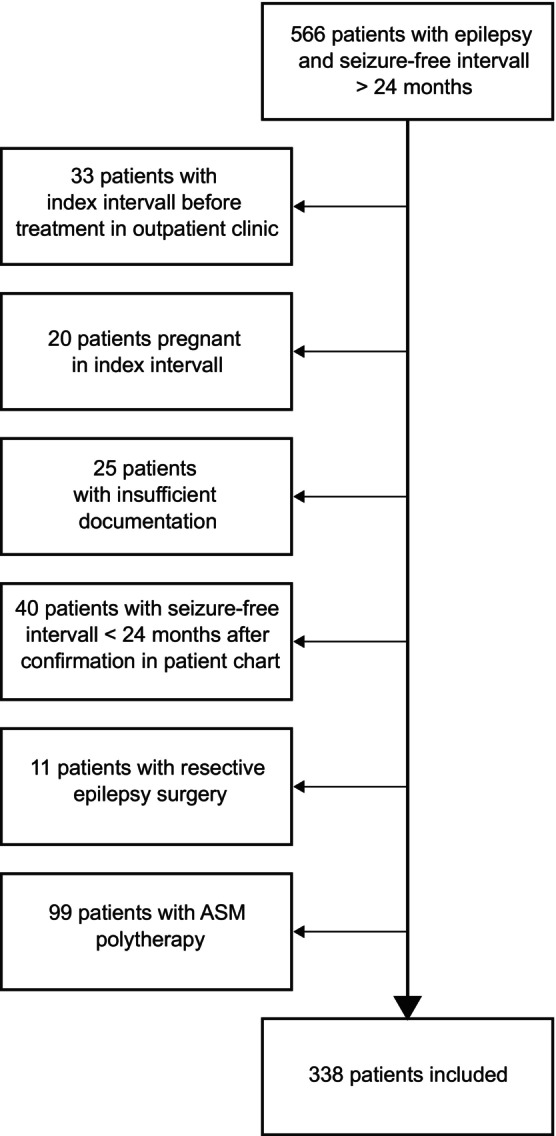
Flow diagram showing the selection of patients for this study.

In all, 566 patients with a seizure‐free interval of ≥24 months were identified. After applying the exclusion criteria, 338 patients were eligible for inclusion in the study. Figure 2 shows the patient selection.

Median age at index visit was 51 years (IQR 36–68); 52.1% were female. Median duration of epilepsy was 17 years (IQR 8–34) and median duration of seizure‐free interval before the index visit was 5 years (IQR 3–10).

### Primary outcomes

In 81.7% of epilepsy patients (*n* = 276), ASM was not discontinued and there was no ASM dose reduction. Accordingly, only in 11.5% (*n* = 39) was ASM discontinued, and the ASM dose was significantly reduced in 6.8% (*n* = 23). Table 1 contrasts different variables in patients in whom ASM was not discontinued. Non‐discontinuation rates for individual ASMs are listed in Table S1 (supplementary material).

**TABLE 1:** Clinical variables associated with non‐discontinuation of ASM therapy.

	Non‐discontinuation of ASM (*n* = 276)	Discontinuation or significant dose reduction of ASM (*n* = 62)	Univariate analysis	Binary logistic regression, exp(*B*) (95% CI)
Female sex, *n* (%)	140 (50.7%)	36 (58.1%)	*p* = 0.296^a^	0.76 (0.41–1.42)
Age, years, median (IQR)	51 (37–68)	51 (33–71)	*p* = 0.804^b^	1.01 (0.99–1.02)
Duration of seizure freedom, years, median (IQR)	5 (3–10)	6 (3–12)	*p* = 0.203^b^	1.00 (0.96–1.04)
Duration of epilepsy until remission, years, median (IQR)	9.5 (1–24)	6 (1–16)	*p* = 0.149^b^	1.01 (0.98–1.03)
Generalized tonic–clonic or focal to bilateral tonic–clonic seizures, *n* (%)	244 (90.0%)	47 (75.8%)	*p* = 0.002^a^	2.33 (1.08–5.06)
Epilepsy type, *n* (%)			*p* = 0.484^a^	Not included^c^
Focal	194 (70.3%)	46 (74.2%)		
Generalized	62 (22.5%)	10 (16.1%)		
Unclassified	20 (7.2%)	6 (9.7%)		
Structural epilepsy, *n* (%)	94 (34.2%)	19 (30.6%)	*p* = 0.594^a^	Not included^c^
History of status epilepticus, *n* (%)	20/230^d^ (8.5%)	3/50^d^ (5.7%)	*p* = 0.529^a^	Not included^c^
First‐generation ASM, *n* (%)	63 (22.8%)	20 (32.3%)	*p* = 0.119^a^	0.78 (0.36–1.67)
History of breakthrough seizure, *n* (%)	57 (20.7%)	4 (6.5%)	*p* = 0.009^a^	3.32 (1.10–10.04)
History of failed attempt to reduce dose or discontinue ASM, *n* (%)	36 (13.0%)	2 (3.2%)	*p* = 0.027^a^	4.67 (1.03–21.11)
Intellectual disability, *n* (%)	15 (5.4%)	4 (6.5%)	*p* = 0.753^a^	Not included^c^
LAEP score, median (IQR)	33 (26–41)	32 (27–40)	*p* = 0.546^b^	Not included^c^
Number of all ASM since diagnosis of epilepsy, median (IQR)	2 (1–3)	1 (1–3)	*p* = 0.088^b^	1.02 (0.79–1.32)
ASM load at index visit, median (IQR)	0.67 (0.5–1)	0.37 (0.33–0.67)	*p* < 0.001^b^	6.10 (2.09–17.78)
Calculated 2‐year recurrence risk, %, median (IQR)	57 (41–70)	50 (37–64)	*p* = 0.058^b^	Not included^e^
Calculated 5‐year recurrence risk, %, median (IQR)	70 (52–81)	62 (47–77)	*p* = 0.060^b^	Not included^e^

Abbreviations: ASM, antiseizure medication; CI, confidence interval; IQR, interquartile range; LAEP, Liverpool Adverse Effects Profile; *n*, number.

^a^ Pearson's chi‐squared test.

^b^ Mann–Whitney *U* test.

^c^ Age and sex as well as variables with *p* values <0.25 in univariate analysis were included in our binary logistic regression model; inclusion method: enter.

^d^ Available data on status epilepticus in 280/338 patients.

^e^ These variables were excluded from the binary logistic regression model to avoid multicollinearity.

In the logistic regression analysis, non‐discontinuation of ASM was independently associated with (i) history of focal to bilateral or generalized tonic–clonic seizures (OR 2.33, 95% CI 1.08–5.06), (ii) history of breakthrough seizures (OR 3.32, 95% CI 1.10–10.04), (iii) history of a failed attempt to discontinue or reduce dose of ASM (OR 4.67, 95% CI 1.03–21.11) and (iv) higher ASM load at the index visit (OR 6.10, 95% CI 2.09–17.78) (Table 1).

**FIGURE 3:**
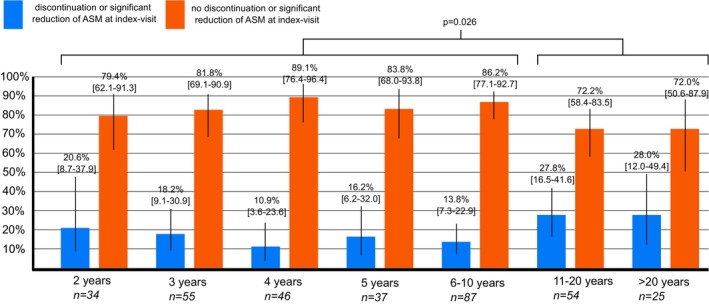
Rate of discontinuation and continuation of ASM therapy according to duration of seizure freedom. This figure shows the rate of patients who continued ASM (red bar) and who discontinued ASM or significantly reduced the dose of ASM (blue bar). Vertical lines represent 95% confidence intervals. ASM, antiseizure medication, *n*, number.

When entered as a continuous variable, there was no statistically significant association between the decision not to discontinue or not to reduce the dose of ASM and duration of seizure freedom. Figure 3 shows discontinuation rates in relation to duration of seizure freedom; a seizure‐free period of ≤10 years was associated with non‐discontinuation of ASM (*p* = 0.026).

### Secondary outcomes

A discussion between physicians and patients regarding discontinuation or dose reduction of ASM was documented in 54.1% (*n* = 183) of index visits.

In 117 out of 276 patients (42.3%) where ASM was not discontinued, documentation was sufficient to evaluate whether this decision was mainly driven by the patient or by the epileptologist. Physicians advised to remain on ASM therapy in 38 cases (32.4%) and patients wished to stay on current ASM in 79 cases (67.5%).

Out of the 62 patients who discontinued or reduced the ASM dose, 35 patients (56.5%) had a follow‐up visit to the outpatient clinic (case example (b) in Figure 1). The median time from the intended discontinuation/dose reduction to last follow‐up visit was 20 months (IQR 9–35). All patients had followed the decision to discontinue or reduce the dose of ASM. At the last follow‐up visit, 62.9% (*n* = 22) of these patients were still seizure‐free. In patients with seizure recurrence, median time from discontinuation to recurrent seizure was 8 months (IQR 3–18).

Patients who were seizure‐free at the last visit had a significantly lower calculated 2‐year and 5‐year seizure recurrence risk than patients where seizures re‐occurred (53% [40%–68%], *p* = 0.017 vs. 62% [47%–78%] and 66% [50%–80%] vs. 75% [58%–89%], *p* = 0.017).

## DISCUSSION

In this study, almost 82% of patients who had been seizure‐free for at least 2 years remained on their ASM in unchanged dose at the last visit to the outpatient clinic. Non‐discontinuation of ASM was independently associated with history of focal to bilateral or generalized tonic–clonic seizures as well as history of previously failed discontinuation or dose reduction attempts, breakthrough seizures and higher ASM drug load.

In contrast to a recent study on a more heterogeneous patient population (including children, adolescents and patients with ASM polytherapy) [8], non‐discontinuation of ASM per se was not associated with shorter duration of seizure freedom, although there was an association between non‐discontinuation of ASM and duration of seizure freedom ≤10 years. Differences in the findings from our study may be explained through different patient populations, as the aforementioned study included children. In children, the time dependence of successful ASM discontinuation with respect to longer duration of seizure freedom is much better established [17]. Furthermore, the mentioned study reports results of a duration of seizure freedom of up to 10 years, whilst our results go beyond 20 years of seizure freedom. We had hypothesized that the rate of non‐discontinuation of ASM would be high initially, decrease with sustained seizure freedom, and then increase again over time, forming a U‐shaped curve. The rationale behind this idea was that, at the beginning of seizure freedom, the risk of discontinuation would be perceived as too high. Later, it would be considered safer to discontinue medication, but there may be a certain point beyond which the decision not to discontinue would become final and irreversible—a point of no return, so to speak. However, our data did not confirm this hypothesis. On the contrary, after being seizure‐free for 10 or 20 years, ASM was discontinued at even higher rates than after shorter periods of seizure freedom. This finding is important as it suggests that discussing ASM discontinuation with seizure‐free patients is always worthwhile.

Our study is the first to show that history of breakthrough seizures and failed discontinuation or reduction attempts are independently associated with non‐discontinuation of ASM. These findings are worthy of further investigation. To our knowledge, there have been no previous reports exploring whether a failed reduction attempt or history of breakthrough seizures is indeed associated with a lower likelihood of remaining seizure‐free after ASM discontinuation [7]. Regarding our final predictor of ASM non‐discontinuation, ASM load at the index visit, the overall low ASM load stands out. This might be interpreted as an expression of the benign course in our patient cohort. Previously published data from our outpatient clinics on patients with focal epilepsies, including those on polytherapy, also demonstrate similarly low values [18].

It is important to recognize that the decision to continue or discontinue ASM is not solely based on rational factors but also depends on the feelings and attitudes of both patients and physicians. As is known from randomized studies, the 1‐ and 2‐year risk of recurrence in patients who discontinue their medication is twice as high as in patients who remain on ASM [19, 20]. Understandably, not every patient and not every physician is willing to take such a risk, especially as seizure relapse may be more burdensome for the patient than continuing low‐dose ASM [20, 21]. Furthermore, there might be several lifestyle‐associated factors that can hinder discontinuation, such as dependence on the permission to drive motorized vehicles. Therefore, remaining on ASM despite being seizure‐free is a completely acceptable decision. It is crucial, however, that this decision is the result of a shared decision‐making process, necessitating a discussion of the pros and cons with the patient. Such a discussion was documented in approximately half of the index visits, confirming the findings of previous studies [8, 14]. Still, our results showing that ASM might be discontinued even after decades of seizure freedom under ASM suggest that there is still much room to improve. Importantly, it is possible that our study, as well as previous studies, underestimate the rate of discussion regarding ASM discontinuation as this item relies on the physician's documentation. However, retrospective assessment is the only way of measuring the rate of discussion regarding ASM therapy as participation in any prospective study on this matter would come with an inherent bias [22]. The significance of a shared decision‐making process is highlighted by our secondary finding that all patients who eventually made the decision to discontinue ASM or reduce its dose followed through with it.

Of all patients with a follow‐up visit after ASM discontinuation, approximately 63% of patients were still seizure‐free during a median follow‐up time of 20 months. These patients had a significantly lower calculated 2‐year and 5‐year seizure recurrence risk according to the antiepileptic drug withdrawal risk retrieval tool [6]. It is reassuring that there was an association between the calculated risk of seizure recurrence and the actual recurrence rate in our study. The antiepileptic drug withdrawal risk retrieval tool has now been validated in three external cohorts and, although there was a trend towards overestimating the risk of recurrence, it has proven to be reliable [5, 23, 24]. It is important to mention that the prediction tool was not routinely used in counseling our patients, and a relevant number of index visits (19.5%) included in this study occurred prior to the publication of the tool.

### LIMITATIONS

Acknowledging the limitations associated with our retrospective study design is crucial. Therefore, a deliberate emphasis was placed on conducting analysis on objectively verifiable variables. Consequently, the reasoning contributing to continuing or discontinuing ASM, as an example, was not examined. In our opinion, a prospective study is necessary to address these aspects adequately. Still, as in every retrospective study, potential source of bias lies in incomplete data documentation.

In our study, only a very small number of patients had electroencephalogram (EEG) examinations at the index visit, as in our outpatient clinics EEGs are not performed routinely at each visit. Therefore, EEG findings were not included in our analysis. Also, whilst it was possible to conduct an investigation regarding a possible association between non‐discontinuation of ASM and type of epilepsy (focal vs. generalized, as well as structural vs. non‐structural), the patient sample size was too small to do so for individual epilepsy syndromes. Finally, even though this study covers three different outpatient clinics in Berlin with heterogeneous patient populations, the generalizability of our findings should be interpreted with caution.

## CONCLUSION

To summarize, our paper conveys two important findings. First, non‐discontinuation of ASM in long‐term seizure freedom is associated with history of focal to bilateral or generalized tonic–clonic seizures, breakthrough seizures and failed attempt to reduce dose of or discontinue ASM as well as higher current ASM load. As the latter three variables are not part of established scores to estimate seizure relapse risk after discontinuation, further studies are in place to investigate if these factors are in fact associated with an increased recurrence risk. In addition, our study expands previous knowledge on the time dependence of seizure duration to the intervals beyond one decade of seizure freedom. These patients had the highest rate of seizure discontinuation attempts. Thus, there does not seem to be a ‘point of no return’ after which ASM will not be discontinued. Our results should encourage physicians to engage in discussions regarding the discontinuation of ASM, even after an extended period of continuous medical therapy.

## AUTHOR CONTRIBUTIONS

**Jakob I. Doerrfuss:** Conceptualization; data curation; investigation; formal analysis; visualization; writing – original draft. **Thea Hüsing:** Investigation; formal analysis; writing – review and editing. **Luise Graf:** Writing – review and editing; formal analysis. **Maria Ilyas‐Feldmann:** Conceptualization; writing – review and editing; supervision. **Martin Holtkamp:** Conceptualization; supervision; resources; writing – review and editing.

## CONFLICT OF INTEREST STATEMENT

JID, TH, LG and MIF declare no conflicts of interest with respect to the research, authorship, and/or publication of this article. MH reports personal fees from Arvelle, Bial, Desitin, Eisai, Jazz Pharma and UCB within the last 3 years, outside the submitted work.

## Supporting information


Table S1.

## Data Availability

The data that support the findings of this study are available from the corresponding author upon reasonable request.
