# Supplementary material for: Non‐discontinuation of antiseizure medication in seizure‐free epilepsy patients
Source: Eur J Neurol. 2023 Nov 28;31(3):e16160. doi: 10.1111/ene.16160 (PMC11235922; doi:10.1111/ene.16160)
Supplement: Supplementary file 1 — Table S1. [file ENE-31-e16160-s001.docx]

**Non-discontinuation of antiseizure medication in seizure-free epilepsy patients**

Jakob I. Doerrfuss, Thea Hüsing, Luise Graf, Maria Ilyas-Feldmann, Martin Holtkamp

**Supplementary Material**

**Table S 1 – Non-discontinuation rates for individual ASM**

| Antiseizure medication | Total | Non-discontinuation  of ASM | Discontinuation or significant dose reduction of ASM |
| --- | --- | --- | --- |
| Brivaracetam, n (%) | 1 | 1 (100) | 0 (0) |
| Carbamazepine, n (%) | 31 | 22 (71) | 9 (29) |
| Eslicarbazepine, n (%) | 13 | 13 (100) | 0 (0) |
| Ethosuximide, n (%) | 1 | 1 (100) | 0 (0) |
| Gabapentin, n (%) | 5 | 4 (80) | 1 (20) |
| Lacosamide, n (%) | 4 | 4 (100) | 0 (0) |
| Lamotrigine, n (%) | 113 | 98 (87) | 15 (13) |
| Levetiracetam, n (%) | 93 | 72 (77) | 21 (23) |
| Oxcarbazepine, n (%) | 12 | 11 (92) | 1 (8) |
| Phenytoin, n (%) | 1 | 1 (100) | 0 (0) |
| Primidone, n (%) | 4 | 4 (100) | 0 (0) |
| Topiramate, n (%) | 10 | 8 (80) | 2 (20) |
| Valproic acid, n (%) | 46 | 35 (76) | 11 (24) |
| Zonisamide, n (%) | 4 | 2 (50) | 2 (50) |

ASM = antiseizure medication, n = number
